# In-depth cerebrovascular lipidomics profiling for discovering novel biomarkers and mechanisms in moyamoya and intracranial atherosclerotic disease

**DOI:** 10.1097/JS9.0000000000002092

**Published:** 2024-09-30

**Authors:** Kangmin He, Xinmei Wang, Yu Gu, Xiao Tong, Xuanfeng Qin, Yujun Liao, Li-Hao Huang, Jiaxi Wang, Bin Xu

**Affiliations:** a Department of Neurosurgery, Fudan University Huashan Hospital, Neurosurgical Institute of Fudan University, Shanghai Clinical Medical Center of Neurosurgery, Shanghai Key Laboratory of Brain Function and Restoration and Neural Regeneration, Shanghai, China; b The Human Phenome Institute, Zhangjiang-Fudan International Innovation Center, Fudan University, Shanghai, China; c State Key Laboratory of Genetic Engineering, Department of Biochemistry and Biophysics, School of Life Sciences, Fudan University, Shanghai, China; d Shanghai Key Laboratory of Metabolic Remodeling and Health, Institute of Metabolism & Integrative Biology, Liver Cancer Institute, Zhongshan Hospital, Fudan University, Shanghai, China

**Keywords:** diagnostic biomarkers, intracranial atherosclerosis disease, iron metabolism, lipidomics, moyamoya disease

## Abstract

**Background:**

Despite considerable research efforts, the precise etiology and underlying pathways contributing to moyamoya disease (MMD) remain poorly understood. Moreover, the overlapping vascular pathologies shared between MMD and intracranial atherosclerotic disease (ICAD) pose challenges in clinical differentiation, even with gold-standard cerebral angiography. An in-depth exploration of lipidomic alterations in cerebral intracranial MMD vessels could offer valuable insights into the pathogenesis of MMD-related mechanisms, encompassing MMD and ICAD, and unveil novel biomarkers and potential therapeutic targets. However, to date, comprehensive lipidomic profiling has been lacking.

**Materials and methods:**

To discover novel biomarkers and unravel the pathophysiological mechanisms underlying MMD, we conducted a lipidomics analysis to characterize various lipid species in matched human extracranial and intracranial artery tissues from patients diagnosed with MMD (*n*=99) and ICAD (*n*=12).

**Results:**

Our analysis identified 569 lipid species and delineated a robust panel of lipidomic biomarkers capable of effectively distinguishing MMD from ICAD (area under curve=0.98), as determined by receiver operating characteristic curve analysis. Notably, we observed a significantly more pronounced positive correlation of diacylglycerols and a negative association of triglycerides in intracranial artery tissues of MMD patients compared to those with ICAD, suggesting a potential role of dysregulated diacylglycerol-induced signaling in MMD pathogenesis. Furthermore, our investigation into the correlations of critical differential intracranial artery vessel lipid species between MMD and ICAD and clinical parameters revealed negative associations with plasma iron levels, implying a potential link between plasma iron metabolism and artery lipid homeostasis during MMD pathogenesis.

**Conclusion:**

These findings offer promising prospects for advancing clinical diagnosis for enhanced differentiation between the two disease conditions. Additionally, they shed light on the fundamental mechanisms implicated in MMD pathogenesis and suggest potential therapeutic avenues through targeting artery vessel lipids or plasma iron levels.

## Introduction

Highlights

- A lipidomic analysis of cerebral arteries in moyamoya disease (MMD) and intracranial atherosclerotic disease (ICAD) patients identified distinct lipidomic biomarkers for distinguishing between the two conditions.
- Intracranial artery tissues from MMD patients exhibited a notable correlation between diacylglycerols and triglycerides, implicating dysregulated diacylglycerol-induced signaling in MMD development.
- Correlations between distinct intracranial artery lipid species in MMD and ICAD patients and clinical parameters unveiled associations with plasma iron levels, implying a potential role in MMD pathogenesis.
- These findings not only improve clinical diagnosis of MMD and ICAD but also indicate potential therapeutic strategies by targeting artery vessel lipids or plasma iron levels for MMD treatment.

Moyamoya disease (MMD) is a cerebrovascular disorder marked by the narrowing of internal carotid arteries, leading to compensatory vessel formation at the brain’s base and severe neurological deficits or even death^1^. There are currently no widely accepted biomarkers for accurately diagnosing MMD or distinguishing it from similar cerebrovascular diseases like intracranial atherosclerotic disease (ICAD) because ICAD shares similar features with MMD^2^. Accurate differentiation is essential for appropriate treatment and understanding of their distinct pathophysiologies.

The unique vascular pathology of MMD involves intimal fibrocellular thickening and smooth muscle cell proliferation, contrasting with the lipid accumulation and monocyte/macrophage infiltration characteristic of ICAD^3–5^. These findings indicate that MMD and ICAD likely involve distinct vessel wall remodeling processes. Despite extensive research, the exact cause remains unknown.

We employed untargeted lipidomics and profiled lipids from human cerebral artery tissues as small as 0.2 cm×0.2 cm from 99 MMD and 12 ICAD patients, analyzing paired intracranial and extracranial artery tissues to identify lipidomic alterations and potential lipid biomarkers. This is the first report of human cerebral artery tissue lipidomics focused on discovering novel lipid biomarkers and elucidating potential mechanisms in MMD and ICAD.

## Methods

### Baseline characteristics of patients

**Figure 1:**
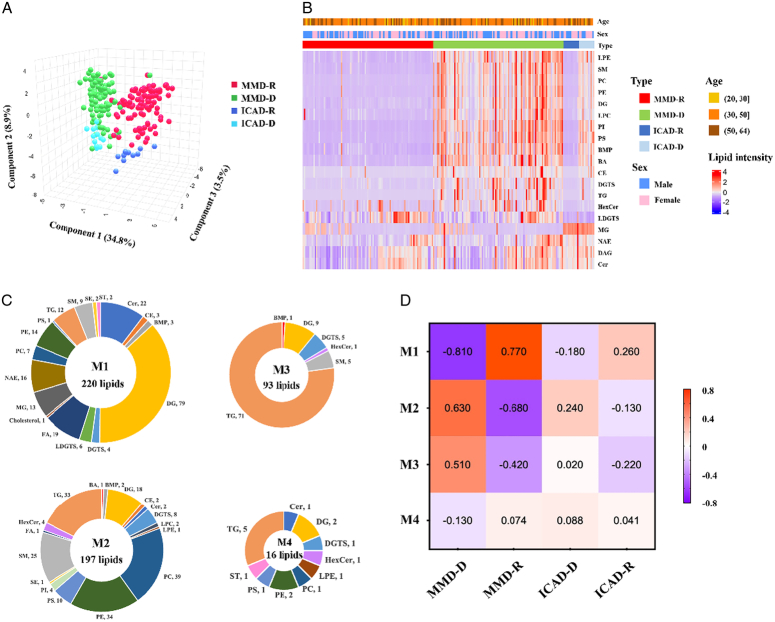
Lipidomics analysis of recipient and donor cerebral vessel tissues in patients with MMS and ICAD. (A) Three-dimensional plot of sparse PLS discriminant analysis of lipidome of the MMD-R, MMD-D, ICAD-R, and ICAD-D groups. (B) Heatmaps showed differences in lipid class levels among tissues of MMD-R, MMD-D, ICAD-R, and ICAD-D. Clinical parameters were plotted corresponding to disease subtypes, including age and sex. (C) WGCNA analysis of lipid profiles generated four lipid modules, including the turquoise module (M1), blue module (M2), brown module (M3), and gray module (M4). (D) The pairwise correlation heatmap between four modules and disease statuses.

Initially, 292 patients were recruited and confirmed with MMD-type cerebrovascular disease via MRI and catheter angiography. Verification was based on stenosis in the terminal internal carotid, proximal middle, and anterior cerebral arteries, with consideration of both unilateral and bilateral involvement. Of these, 83 patients were excluded due to alternative diagnoses: middle artery occlusion (*n*=55), carotid artery occlusion (*n*=9), nasopharyngeal carcinoma (*n*=7), aneurysm (*n*=4), cerebral microvascular disease (*n*=6), cystic acoustic neuroma (*n*=1), and arteriovenous malformation (*n*=1). The remaining 209 patients included 18 with ICAD and 191 with MMD. For lipidomics analysis, we selected patients with paired recipient intracranial and donor extracranial artery vessels and excluded those with insufficient sample sizes (less than 0.2 cm×0.2 cm) in either vessel. This resulted in a final cohort of 111 patients: 12 with ICAD and 99 with MMD, from whom we profiled lipid species in artery tissues (see Fig. 1).

## Results

### Demographic characteristics of patients with MMD and ICAD

We collected a cohort of 111 patients, comprising 12 ICAD patients and 99 MMD patients with intact paired donor and recipient artery vessels for lipidomic analysis. (Supplementary Fig. S1, Supplemental Digital Content 1, http://links.lww.com/JS9/D496). Comparative analysis showed no significant differences in age or gender between MMD and ICAD groups, but variations in clinical plasma parameters indicated differences in iron metabolism and inflammatory responses (Supplementary Table S1, Supplemental Digital Content 1, http://links.lww.com/JS9/D496). Using the LASSO regression algorithm, we developed a risk score system from the training set (Supplementary Fig. S2A, B, Supplemental Digital Content 1, http://links.lww.com/JS9/D496). This score was significantly higher in the MMD group than ICAD (*P*<0.0001, Supplementary Fig. 2C, Supplemental Digital Content 1, http://links.lww.com/JS9/D496), suggesting that clinical parameters related to immune activation and inflammation can differentiate between the two conditions. However, challenges remain in predicting MMD pathogenesis, as systemic inflammation is present in ICAD but not in MMD.

### Lipidomics of intracranial and extracranial cerebral vessels from MMD and ICAD patients

Using untargeted lipidomics, we profiled vessel tissues from four sources: MMD extracranial donor cerebral vessels (MMD-D), MMD intracranial recipient cerebral vessels (MMD-R), ICAD extracranial donor cerebral vessels (ICAD-D), and ICAD intracranial recipient cerebral vessels (ICAD-R) (Supplementary Fig. S3, Supplemental Digital Content 1, http://links.lww.com/JS9/D496). A total of 569 lipid species were identified, spanning various classes like ceramides, cholesterol, triglycerides, and phospholipids. A three-dimensional PLS-DA plot distinctly separated the lipidomes of the four groups (Fig. 1A). Heatmap integration of lipid classes with clinical parameters revealed distinct expression patterns despite non-significant age and sex differences (Fig. 1B). Cumulative lipid composition and violin plots highlighted higher lipid concentrations in donor versus recipient tissues and distinct lipid profiles in intracranial vessels (Fig. 1C, Supplementary Figs. S4, S5, Supplemental Digital Content 1, http://links.lww.com/JS9/D496). WGCNA identified four lipid modules (M1–M4). M1, rich in diacylglycerol (DG), showed a strong correlation with MMD-R and weaker with ICAD-R and was linked to lipid signaling (Fig. 1D, Supplementary Fig. S6, Supplemental Digital Content 1, http://links.lww.com/JS9/D496). M2 and M3, enriched in triglycerides (TG), correlated with MMD-D and ICAD-D, associated with membrane components and lipid storage (Supplementary Figs. S7, S8, Supplemental Digital Content 1, http://links.lww.com/JS9/D496). The positive DG and negative TG correlations in MMD-R versus MMD-D, absent in ICAD, suggest distinct pathogenic mechanisms between MMD and ICAD.

### The lipidomic comparison between intracranial and extracranial cerebral vessels in MMD patients

Following WGCNA analysis, we compared lipid species between MMD-D and MMD-R in MMD patients, revealing distinct separation via two-dimensional principal component analysis (PCA) (Supplementary Fig. S9A, Supplemental Digital Content 1, http://links.lww.com/JS9/D496). The MMD-R group showed significantly lower total lipid content than MMD-D (Supplementary Figs. S4, S5, Supplemental Digital Content 1, http://links.lww.com/JS9/D496). A heatmap (Supplementary Fig. S9B, Supplemental Digital Content 1, http://links.lww.com/JS9/D496) and volcano plot (Supplementary Fig. S9C, Supplemental Digital Content 1, http://links.lww.com/JS9/D496) identified 292 differential lipids (fold change >2, *P*<0.05), with 126 upregulated and 166 downregulated species in MMD-R. Notably, TG and DG exhibited significant dysregulation, highlighting lipid alterations in the intracranial artery where MMD pathology manifests. Chord diagrams of lipid correlations in MMD-D (Supplementary Fig. S9D, Supplemental Digital Content 1, http://links.lww.com/JS9/D496) and MMD-R (Supplementary Fig. S9E, Supplemental Digital Content 1, http://links.lww.com/JS9/D496) showed weaker correlations in the recipient artery vessels, with DGs upregulated and TGs downregulated in MMD-R (Supplementary Figs. S10, S13A, Supplemental Digital Content 1, http://links.lww.com/JS9/D496).

### The lipidomic comparison between intracranial and extracranial cerebral vessels in ICAD patients

Similarly, we compared lipid species between ICAD-D and ICAD-R in ICAD patients using PCA (Supplementary Fig. S11A, Supplemental Digital Content 1, http://links.lww.com/JS9/D496). The lipid content in the ICAD-R group was notably lower than in the ICAD-D group (Supplementary Figs. S4, S5, Supplemental Digital Content 1, http://links.lww.com/JS9/D496). A heatmap (Supplementary Fig. S11B, Supplemental Digital Content 1, http://links.lww.com/JS9/D496) and volcano plot (Supplementary Fig. S11C, Supplemental Digital Content 1, http://links.lww.com/JS9/D496) identified 295 differential lipids (fold change >2, *P*<0.05), with 130 upregulated and 165 downregulated species in ICAD-R. Chord diagrams of lipid species intercorrelations in ICAD-D (Supplementary Fig. S11D, Supplemental Digital Content 1, http://links.lww.com/JS9/D496) and ICAD-R (Supplementary E, Supplemental Digital Content 1, http://links.lww.com/JS9/D496) revealed weaker correlations than those observed in MMD vessels. Surprisingly, the downregulation of TGs and upregulation of DGs seen in MMD-R compared to MMD-D were less pronounced in ICAD vessels (Supplementary Figs. S10, S12, S13, Supplemental Digital Content 1, http://links.lww.com/JS9/D496).

### Differential lipid species between the intracranial and extracranial artery tissues in predicting MMD and ICAD

**Figure 2:**
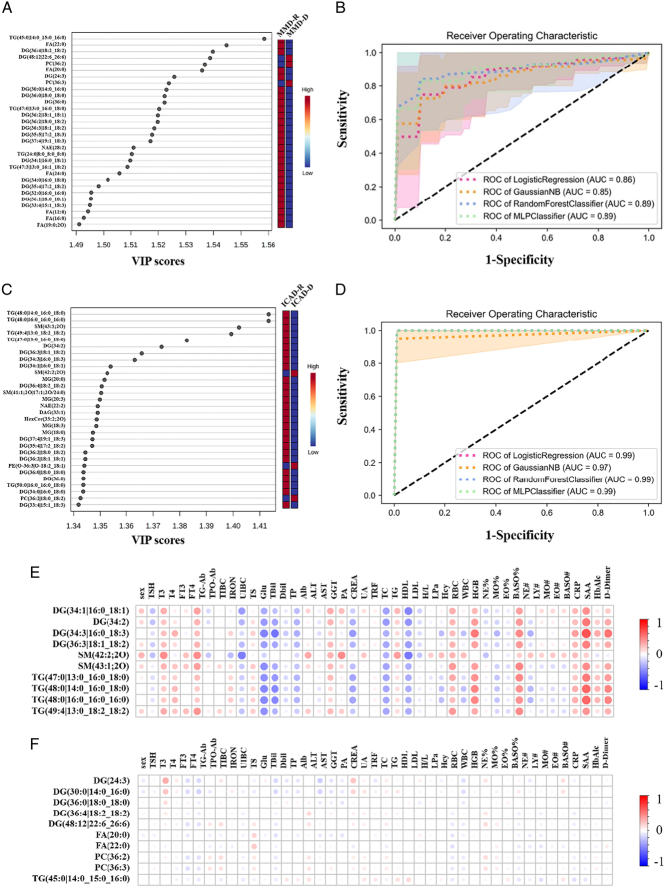
Association of differential lipid species of recipient and donor cerebral vessel tissues with clinical parameters. The top 30 lipid species ranged by VIP (variable importance on projection) score, differentiating (A) MMD-R from MMD-D and (C) ICAD-R from ICAD-D, applying stringent criteria (*P*<0.05, fold change >2, and VIP score >1). Receiver operating characteristic curves generated by four machine learning algorithms, including logistic regression, Gaussian NB, random forests, and MLP classifier differentiating (B) MMD-R from MMD-D and (D) ICAD-R from ICAD-D. The shade indicates the results of 10-fold cross-validation. Association of clinical parameters with differential lipid species between (E) ICAD-R and ICAD-D and (F) MMD-R and MMD-D.

We explored whether lipid species could serve as predictive biomarkers for MMD and ICAD. Using the top 10 lipid species ranked by variable importance in projection (VIP) (Fig. 2A), we applied machine learning algorithms to differentiate between MMD-R and MMD-D groups, achieving high AUC values in ROC curves (Fig. 2B). However, these lipid species showed no significant correlation with clinical parameters in MMD, highlighting the potential of lipidomic profiles over current biomarkers (Fig. 2F). Similarly, the top 10 differential lipid species between ICAD-R and ICAD-D demonstrated strong performance in machine learning models (Fig. 2C), with AUC values up to 0.99 (Fig. 2D). Unlike in MMD, these lipid species in ICAD correlated positively with clinical parameters related to systemic inflammation and metabolic disorders. This suggests that ICAD lipid profiles reflect systemic conditions (Fig. 2E), whereas MMD’s complexity may require more specific diagnostic approaches beyond clinical parameters.

### Differentiation of MMD and ICAD and associations of clinical parameters with differential lipid species between intracranial MMD and ICAD recipient artery tissues

**Figure 3:**
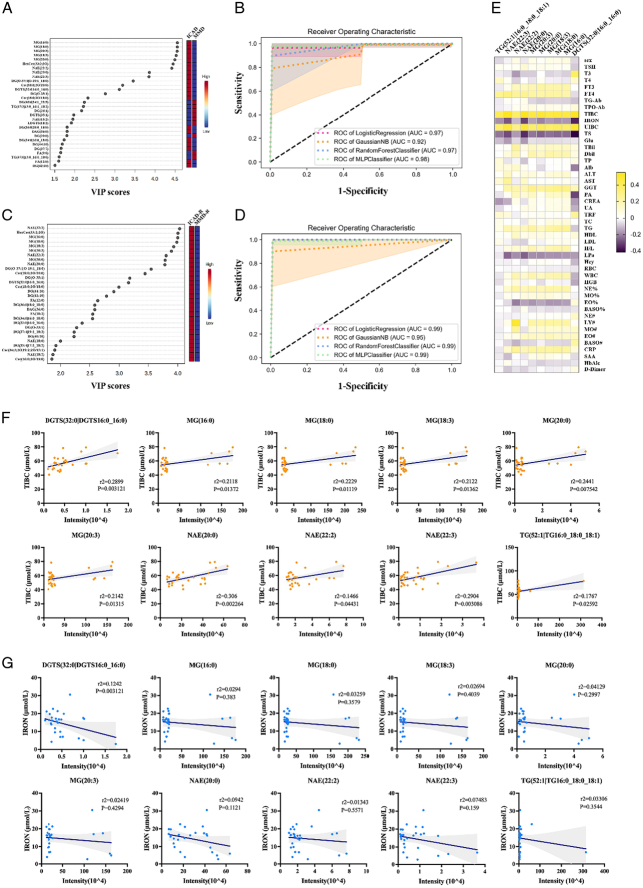
Association of differential lipid species between intracranial MMD and ICAD recipient artery tissues with clinical parameters. The top 30 lipid species ranged by VIP (variable importance on projection) score, differentiating (A) MMD-D from ICAD-D and (C) MMD-R from ICAD-R. Ten lipid species were selected based on stringent criteria (*P*<0.1, fold change >2, and VIP score >1) to distinguish (B) MMD-D from ICAD-D, including MG (16:0), MG (18:3), MG (18:0), NAE (22:2), NAE (22:3), MG (20:3), MG (20:0), NAE (20:0), TG (52:1|16:0_18:0_18:1), and PE (34:2), and (C) MMD-R from ICAD-R, including NAE (22:3), MG (16:0), MG (18:3), MG (20:3), NAE (22:2), MG (18:0), MG (20:0), DGTS (32:0|16:0_16:0), NAE (20:0), and TG (52:1|16:0_18:0_18:1), by receiver operating characteristic curves generated by four machine learning algorithms, including logistic regression, Gaussian NB, random forests, and MLP classifier differentiating. (E) Association of clinical parameters with differential lipid species between MMD-R and ICAD-R. (F and G) Linear regression analysis of TIBC and IRON abundance (μmol/l) and differential lipid species.

We investigated whether arterial lipid species could serve as predictive biomarkers for differentiating MMD and ICAD. The top 10 lipid species, selected based on VIP rank (Fig. 3A), were applied in machine learning algorithms, demonstrating high AUC values in ROC curves for both conditions (Fig. 3B and Supplementary Figs. S10, S12, Supplemental Digital Content 1, http://links.lww.com/JS9/D496). Further analysis utilized lipid species selected from significant differences in intracranial artery between MMD and ICAD (Fig. 3C, D) revealed strong positive correlations between those lipid species and clinical parameters like TIBC and UIBC in MMD patients (Fig. 3E, F, and Supplementary Fig. S14A, Supplemental Digital Content 1, http://links.lww.com/JS9/D496), alongside negative correlations with iron and TS levels (Fig. 3G and Supplementary Fig. S14B, Supplemental Digital Content 1, http://links.lww.com/JS9/D496). These findings suggest that lipidomic profiles could enhance MMD diagnostics and highlight a potential link between iron deficiency and MMD pathogenesis.

## Discussion

In this study, we aimed to profile the lipidomics of MMD and ICAD vessels, marking the first comprehensive examination of human pathological and normal artery lipidomics in these conditions. We identified a robust panel of lipidomic biomarkers capable of differentiating intracranial and extracranial arterial vessels and effectively predicting MMD and ICAD. In MMD but not ICAD patients, we observed more pronounced upregulation of DG species and downregulation of TG species in intracranial recipient arteries compared to extracranial donor vessels. The accumulation of DG, rather than TG, in MMD vessels may explain the unique vascular pathology of MMD, which contrasts with the atherosclerotic progression seen in ICAD, characterized by higher TG levels and macrophage-driven lipid droplet formation^6^. The accumulated DG species may regulate signaling pathways involved in angiogenesis, further distinguishing MMD from ICAD^7^.

In addition, the top differential lipidomic biomarkers between MMD and ICAD showed negative correlations with plasma iron levels, suggesting a link between iron metabolism and artery lipid homeostasis in MMD pathogenesis. Iron deficiency can create a proangiogenic microenvironment via vascular endothelial growth factor, which may enhance the angiogenic potential of MMD vessels^8,9^. Severe iron deficiency anemia can impact artery flow patterns, potentially contributing to intimal hyperplasia and vessel wall ischemia^10^. Further investigation is needed to determine whether iron deficiency contributes to MMD-specific vascular changes distinct from ICAD.

While our study has made significant advancements, certain limitations remain persist. The sample size limits gender-based analyses and correlations with disease severity or subtypes. The lack of family history data, BMI, risk factors, genome-wide associations, and hemodynamic measurements hinders comprehensive correlations with artery lipidomics. Larger, multicenter studies are needed to validate our results and further investigate how these factors correlate with lipidomic profiles in MMD and ICAD.

## Conclusion

In summary, our results revealed a novel panel of arterial tissue lipid biomarkers with high sensitivity and accuracy in distinguishing between MMD and ICAD. Additionally, we observed significant upregulation of DG species and downregulation of TG species when comparing intracranial arteries with extracranial arteries in patients with MMD but not in those with ICAD. Furthermore, the top differential lipidomic biomarkers in intracranial arterial vessels exhibited negative correlations with plasma iron levels. Our findings could advance pathophysiology-based vascular research and support the development of diagnostic and predictive models for vascular diseases (Supplementary Fig. S15, Supplemental Digital Content 1, http://links.lww.com/JS9/D496).

## Ethical approval

This research received approval from the Ethics Committee of Huashan Hospital, Fudan University, under reference number KY2022-1047, and adhered to the principles outlined in the Declaration of Helsinki.

## Consent

Written informed consent was obtained from the patient for publication of this work. A copy of the written consent is available for review by the Editor-in-Chief of this journal on request.

## Source of funding

This work was supported by the ‘Research on Prevention and Treatment of Common Multiple Diseases’ in the 14th Five Year Plan of China, a Key Project of the National Key R&D Program of China (2021YFC2501100), and the National Natural Science Foundation of China (32171168, 32371243, 32350610248).

## Author contribution

K.H.: investigated, wrote, and edited the work; X.W.: investigated and performed the formal analysis; Y.G. and X.Q.: collected samples; X.T.: performed data curation; Y.L.: validated and edited the work; L.H.: conceptualized, supervised and reviewed the work, and worked on the methodology; J.W.: supervised the work and performed data analysis; B.X.: supervised, reviewed the work, and provided resources.

## Conflicts of interest disclosure

The authors report no conflicts of interest.

## Research registration unique identifying number (UIN)

None.

## Guarantor

Bin Xu.

## Data availability statement

The data that support the findings of this study are available from the corresponding author on reasonable request.

## Provenance and peer review

Not commissioned, externally peer-reviewed.

## Supplementary Material
