# Supplementary material for: In-depth cerebrovascular lipidomics profiling for discovering novel biomarkers and mechanisms in moyamoya and intracranial atherosclerotic disease
Source: Int J Surg. 2024 Sep 30;111(1):1607–13. doi: 10.1097/JS9.0000000000002092 (PMC11745776; doi:10.1097/JS9.0000000000002092)
Supplement: Supplementary file 1 [file js9-111-1607-s001.docx]

Supplementary Material

**In-depth cerebrovascular lipidomics profiling for discovering novel biomarkers and mechanisms in moyamoya and intracranial atherosclerotic disease**

Kangmin He, MD^a^, Xinmei Wang, BSc^b,c^, Yu Gu, BSc^d^, Xiao Tong, MSc^d^, Xuanfeng Qin, MD^a^, Yujun Liao, MD^a,*^, Li-Hao Huang, PhD^d,*^, Jiaxi Wang, PhD^b,*^, Bin Xu, MD^a,*^

^a^ Department of Neurosurgery, Fudan University Huashan Hospital, Neurosurgical Institute of Fudan University, Shanghai Clinical Medical Center of Neurosurgery, Shanghai Key Laboratory of Brain Function and Restoration and Neural Regeneration, Shanghai 200040, China.

^b^ The Human Phenome Institute, Zhangjiang-Fudan International Innovation Center, Fudan University, Shanghai 200438, China.

^c^ State Key Laboratory of Genetic Engineering, Department of Biochemistry and Biophysics, School of Life Sciences, Fudan University, Shanghai 200438, China.

^d^ Shanghai Key Laboratory of Metabolic Remodeling and Health, Institute of Metabolism & Integrative Biology, Liver Cancer Institute, Zhongshan Hospital, Fudan University, Shanghai 200438, China.

K. He, X. Wang and Y. Gu contributed equally to this work as co-first authors.

**Corresponding author**

Address: Department of Neurosurgery, Fudan University Huashan Hospital, Neurosurgical Institute of Fudan University, Shanghai Clinical Medical Center of Neurosurgery, Shanghai Key Laboratory of Brain Function and Restoration and Neural Regeneration, No. 12 Middle Wulumuqi Road, Shanghai, 200040, China. Tel.: + 86 135 0170 8779. E-mail: xubinky@sina.com (X. Bin), nsliaoyujun@126.com (Y. Liao) and The Human Phenome Institute, Zhangjiang-Fudan International Innovation Center, Fudan University, No. 2005 Songhu Road, Shanghai, 200438, China. Tel.: + 86 021 3124 2082. E-mail: jiaxiwang@fudan.edu.cn (J. Wang), and Shanghai Key Laboratory of Metabolic Remodeling and Health, Institute of Metabolism & Integrative Biology, Liver Cancer Institute, Zhongshan Hospital, Fudan University, Shanghai 200438, China. Tel.: +861 337 198 9057. E-mail: lihao_huang@fudan.edu.cn (L.-H. Huang).

**Supplementary Methods**

***Artery Tissue Collection***

The study meticulously collected pathological specimens, measuring 0.2 cm by 0.2 cm, from recipient intracranial and donor extracranial artery vessels obtained from the same patients diagnosed with MMD and ICAD. These specimens underwent a rigorous process involving meticulous washing with low-temperature phosphate-buffered saline to remove residual blood cells from the vessels. Following this, the processed artery tissues were carefully preserved at -80 °C, ensuring their integrity for subsequent detailed and comprehensive lipidomics analysis by liquid chromatography-mass spectrometry (LC-MS/MS).

***Untargeted Lipidomics Analysis***

The lipid profiles within vascular tissues were assessed using an untargeted lipidomics methodology primarily based on LC-MS/MS. To extract the lipids, the tissues underwent a specific procedure: they were smashed and combined with 200 μL of PBS in a high-throughput tissue grinder (Wonbio) for three intervals of 30 seconds at 60 Hz. Subsequently, a mixture of 1.5 mL of methanol and 5 mL of MTBE was added and agitated for an hour at room temperature using a shaker. Following this, 1.25 mL of water was introduced and mixed using a vortexer for one minute. After centrifugation at 1000 × g for 10 minutes at 4 ℃, clear stratification of the sample was observed. The supernatant was collected and subjected to drying via a SpeedVac (LABCONCO Refrigerated CentriVap Concentrator). The resulting powder was reconstituted using 80 μL of a redissolution reagent containing 65% acetonitrile, 30% isopropanol, and 5% water. This solution was then centrifuged at 14000 × g for 10 minutes at 4 ℃, and the resulting supernatant was carefully transferred to autosampler vials for subsequent LC-MS analysis. The LC-MS analysis was carried out using a Liquid Chromatography Mass Spectrometry system (AB SCIEX ZenoTOF 7600). Additionally, a quality control (QC) sample was generated by pooling an equal volume of each individual sample, allowing for the assessment of LC-MS analysis reproducibility.

***Data Processing and Statistical Analysis***

For the lipidomics analysis, peak areas of identified lipids were normalized to the total peak area within each sample, a crucial step that mitigated variations introduced during sample handling and instrumental analysis. This normalization process not only ensured consistency across samples but also facilitated direct comparison of lipid abundances. The normalized data were then subjected to a series of statistical analyses. Univariate analyses included t-tests, Mann-Whitney test for quantitative variables, and chi-square test for qualitative variables, with assumptions of normality and homoscedasticity rigorously validated. Multivariate analyses were performed using tools like MetaboAnalyst (v5.0), renowned for its user-friendly interface and comprehensive suite of statistical models, and the LINT website, which provided specialized lipid-focused analytical tools. Visualization of the data was enhanced through techniques such as sparse partial least squares discriminant analysis (sPLS-DA), principal component analysis (PCA), and hierarchical clustering heatmaps, revealing distinct patterns and differences between groups. Volcano plots, generated using the ggplot2 package in R (v4.1.2), offered a dynamic representation of significantly altered lipids, highlighting both the magnitude and statistical significance of changes. Differential metabolites were considered significant with fold change >2 and p <0.05. Correlation analysis and chord diagrams, executed via the "Wu Kong" platform (https://www.omicsolution.com/wkomics/main/), provided a visual representation of the intricate relationships among lipid species. Enrichment analysis was carried out using the LION website (http://lipidontology.com/) in target-list mode, enriching understanding through functional categorization.

Advanced statistical methods were employed for deeper insights. In Lasso regression analysis, all available clinical variables were initially included in the model, with Lasso regression’s feature selection capability identifying only those with significant coefficients. The model was refined by cross-validation, retaining the most influential clinical variables and enhancing predictive accuracy and generalizability for MMD and ICAD risk factors.

The lipid modules were created following the weighted gene correlation network analysis (WGCNA) protocol as implemented in the WGCNA package in R (v4.1.2). The soft thresholding power was determined to ensure a scale-free network topology, essential for constructing the co-expression network, upon which adjacency matrices were computed using Pearson’s correlation coefficients to quantify the correlation between lipid pairs. To organize the lipids into coherent modules, the average linkage hierarchical clustering method was employed to group lipids based on their topological overlap measure (TOM), a metric that captures the shared pattern of connections with all other lipids in the network. A minimum module size of 30 was set to ensure that each module was biologically meaningful and statistically robust. Modules that exhibited a high degree of similarity, as determined by a TOM threshold of 0.25, were merged to form consolidated clusters. This merging process enhanced the interpretability of the network by reducing complexity while preserving biologically relevant groupings. The resulting network was visualized in Cytoscape 3.9.0 using edge and node list files exported from the analysis.

The machine learning model was developed using Python 3.10 by first dividing the dataset into training and testing sets in a 3:1 ratio. Models such as logistic regression, Gaussian Naive Bayes (gaussianNB), random forest, and multi-layer perceptron (MLP) were constructed using tenfold cross-validation within the training set. The classification capabilities were then assessed on the testing set through receiver operating characteristic (ROC) curves, with the 95% confidence interval (CI) calculated from the tenfold cross-validation results. The area under the curve (AUC) was calculated for these ROC curves, providing a quantitative measure of the models’ ability.

**Supplementary Figures**

**Supplementary Figure 1.** A flowchart showing the process of patient selection.


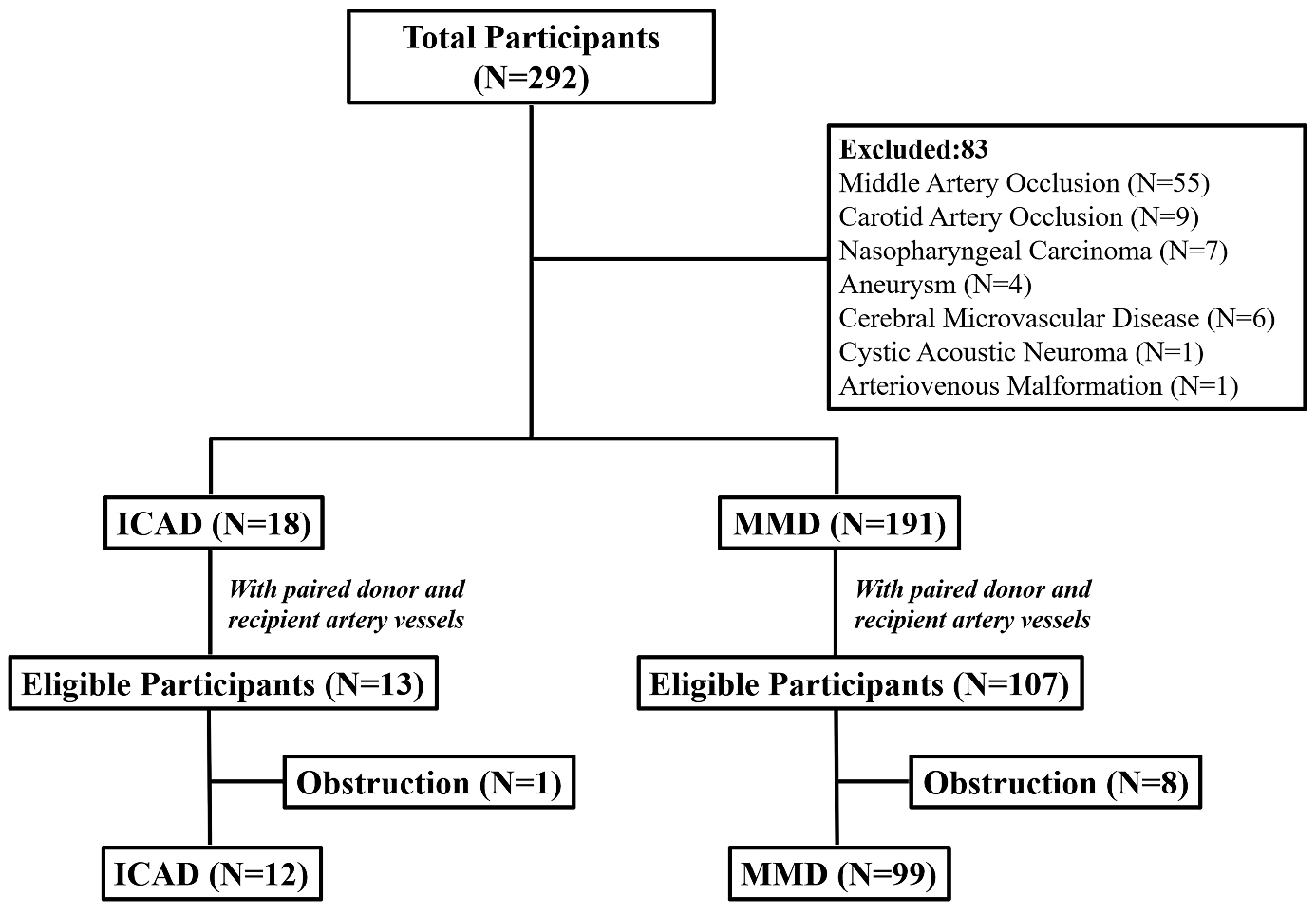


**Supplementary Figure 2.** Construction of Moyamoya disease risk model. Dividing the participants into training and testing sets, the clinical parameters from the training set were used to construct a risk score system via LASSO regression. (A) LASSO deviance profiles and (B) LASSO coefficient profiles. 8 clinical parameters were identified as the most significant contributors to the MMD risk score, including FT3 (coefficient=0.2864), thyroid peroxidase antibodies (TPO-Ab) (coefficient=-0.0001), total bilirubin (Tbill) (coefficient=-0.0700), plasma aldosterone (PA) (coefficient=-0.0004), CREA (coefficient=-0.0021), TRF (coefficient=0.0598), monocyte number (MO#) (coefficient=-0.0495), and CRP (coefficient=-0.0210). (C) Levels of risk score between MMD and ICAD groups. **** p < 0.0001.

**
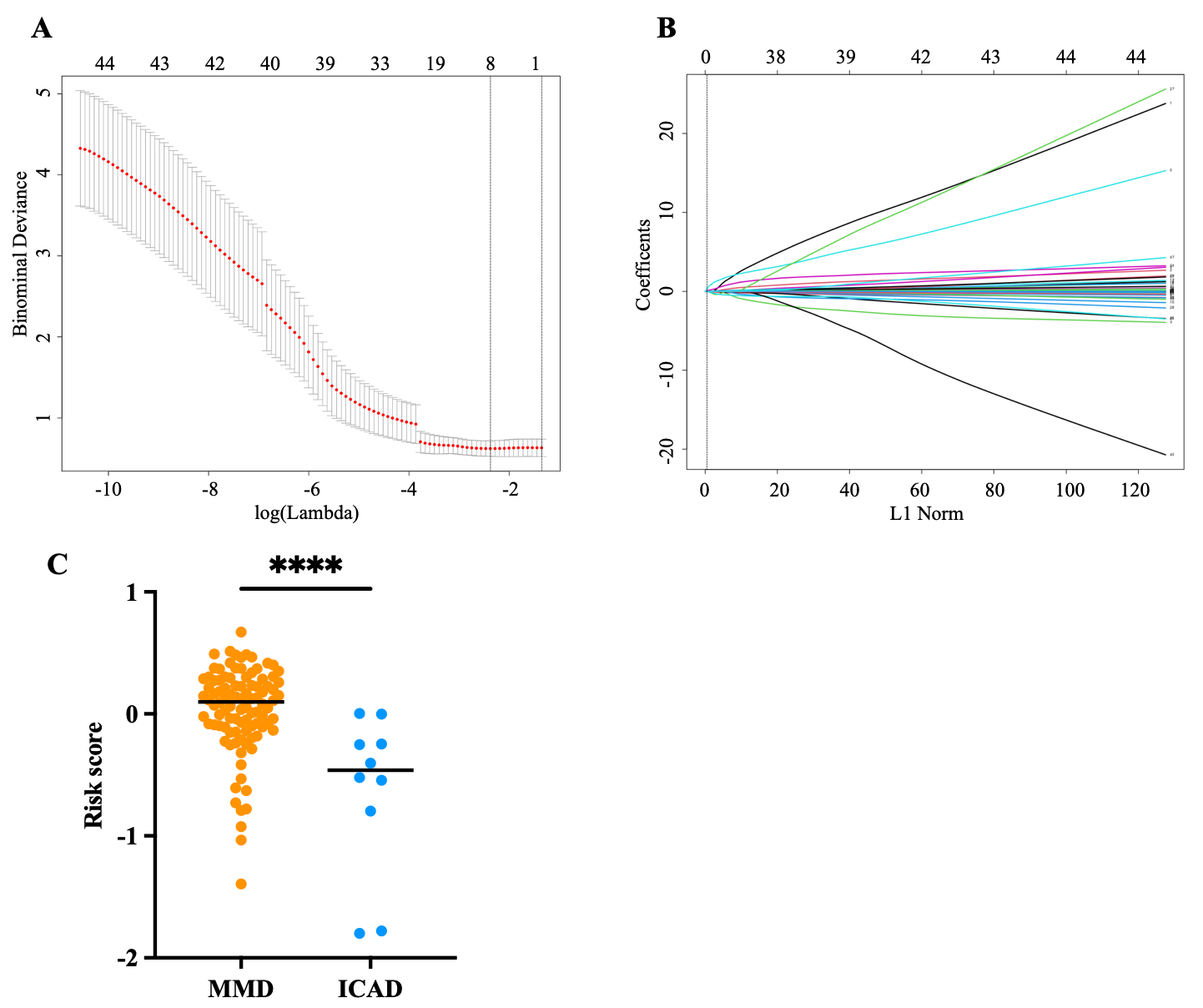
**

**Supplementary Figure 3.** The flowchart of lipidomics analysis based on human brain vessel tissues.


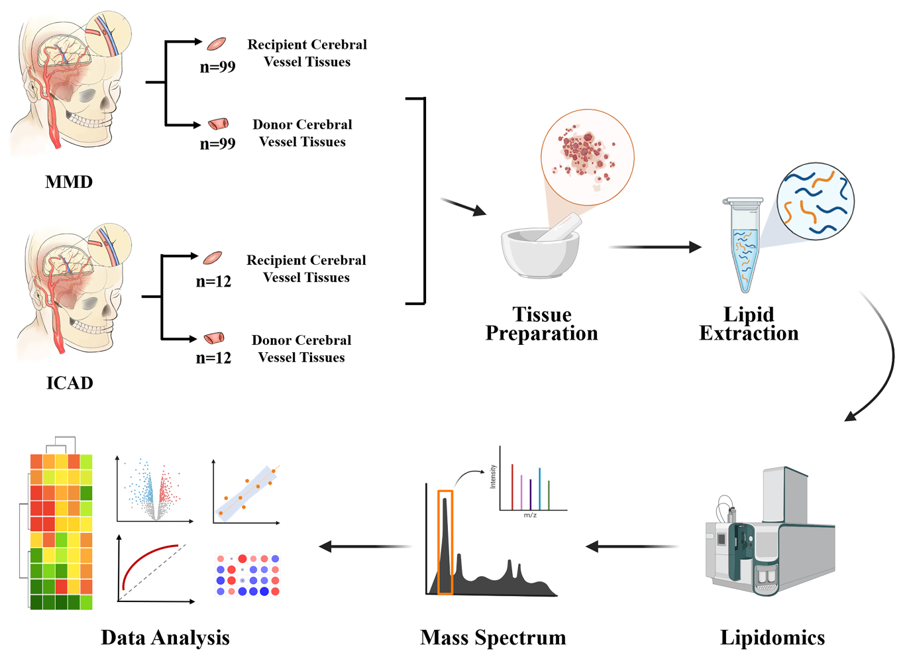


**Supplementary Figure 4.** Cumulative lipid composition of MMD-R, MMD-D, ICAD-R and ICAD-D groups.

******Supplementary Figure 5.** Differences of lipid classes between donor and recipient cerebral vessel tissues in patients with MMD and ICAD. * p<0.05; ** p<0.01; *** p<0.001; **** p<0.0001.


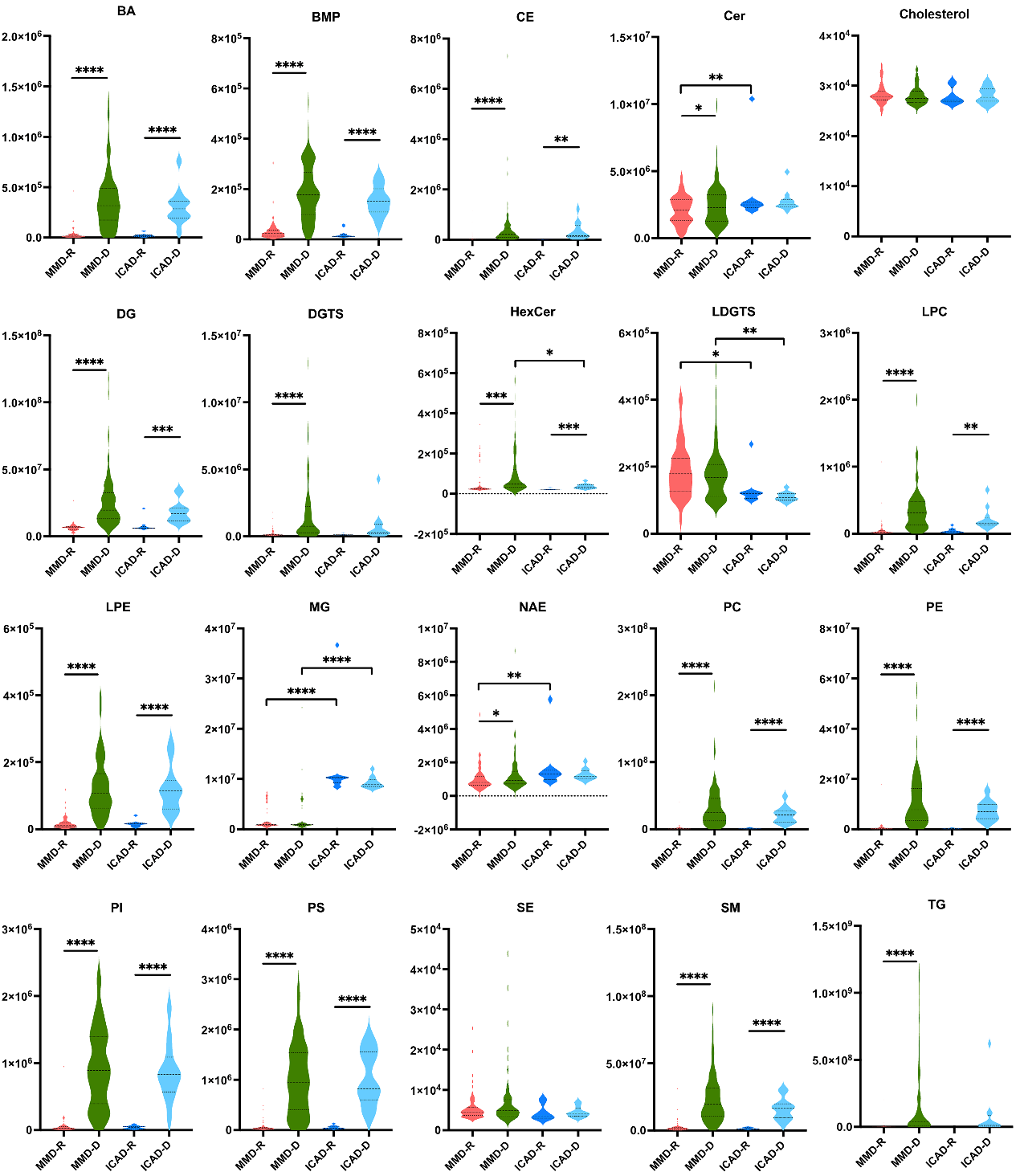


**Supplementary Figure 6.** Enrichment analysis of lipids in M1 module.


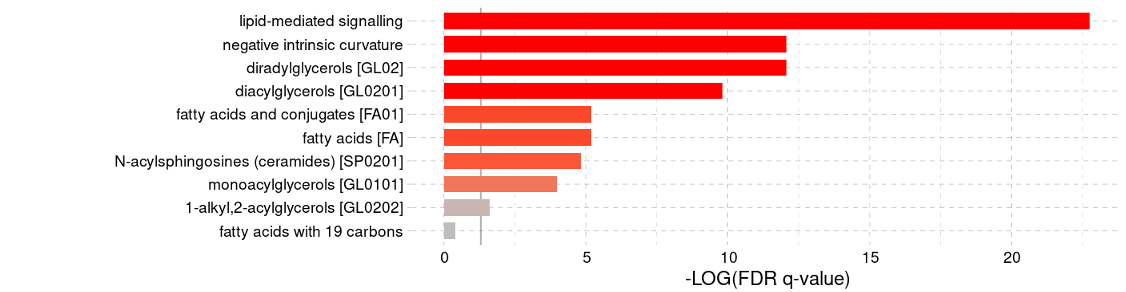


**Supplementary Figure 7.** Enrichment analysis of lipids in M2 module.


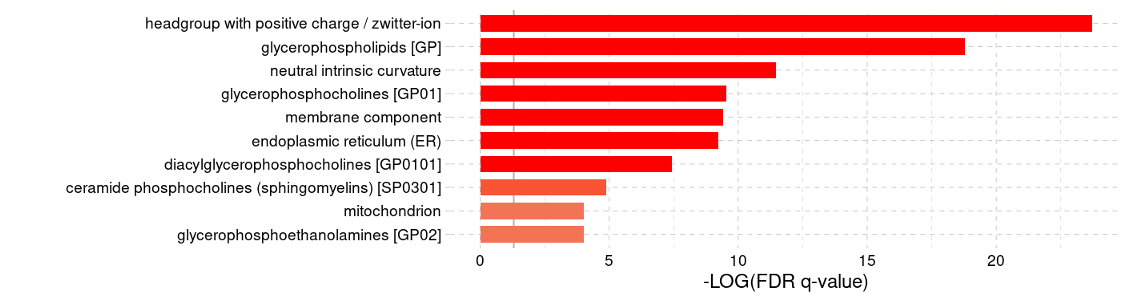


**Supplementary Figure 8.** Enrichment analysis of lipids in M3 module.


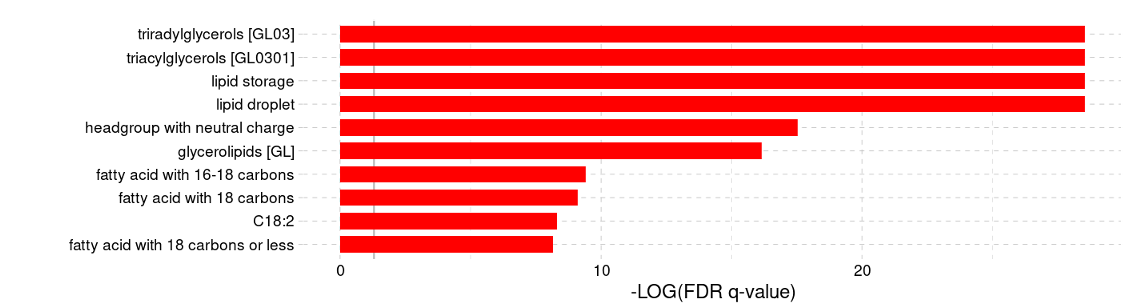


**Supplementary Figure 9.** Distinct lipidomic profiles between MMD-D and MMD-R. (A) Two-dimensional PCA (principal components analysis) plot of lipid profiles of MMD-D and MMD-R groups. (B) The heatmap showed the top 75 differential lipid species between MMD-D and MMD-R groups. (C) Volcano plot of the differences in lipid profiles between MMD-D and MMD-R groups.The lipid correlations between lipid classes of (D) MMD-D and (E) MMD-R groups. Data were log-transformed, and correlations between lipids were calculated using Spearman's correlation analysis. The width of the band indicates the number of correlations, and the color indicates the direction of the correlation. The correlation coefficient cut-off was set to ≥0.2.


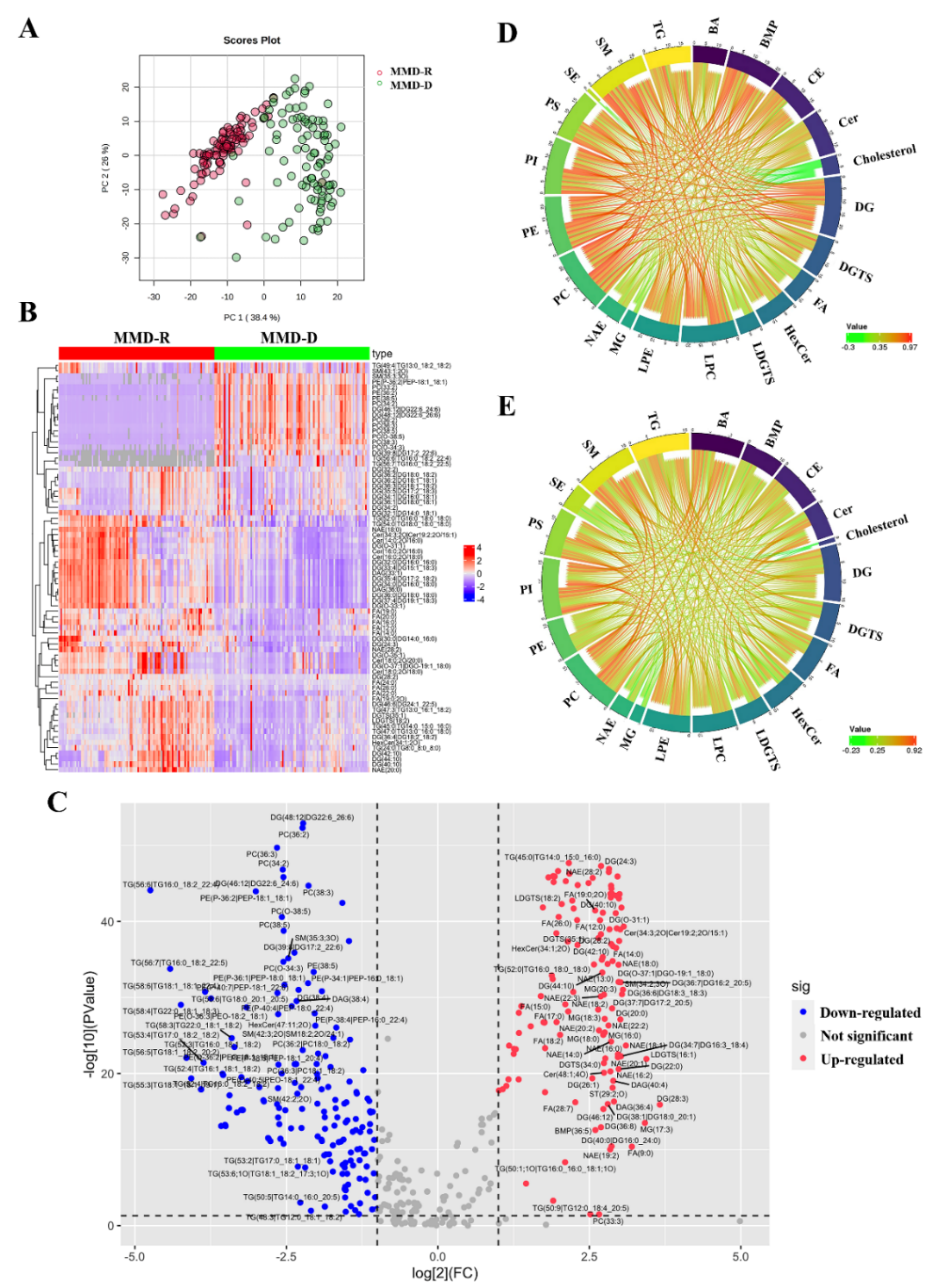


**Supplementary Figure 10.** Differential lipid species between recipient and donor cerebral vessel tissues in patients with MMD. Lipid species within each lipid class are depicted by filled circles and arranged according to the number of total carbon atoms (x axes) and number of unsaturated bonds (y axes). The colors of the circles indicate different fold changes (recipient/donor), while the sizes of the circle indicate different ranges of P values.


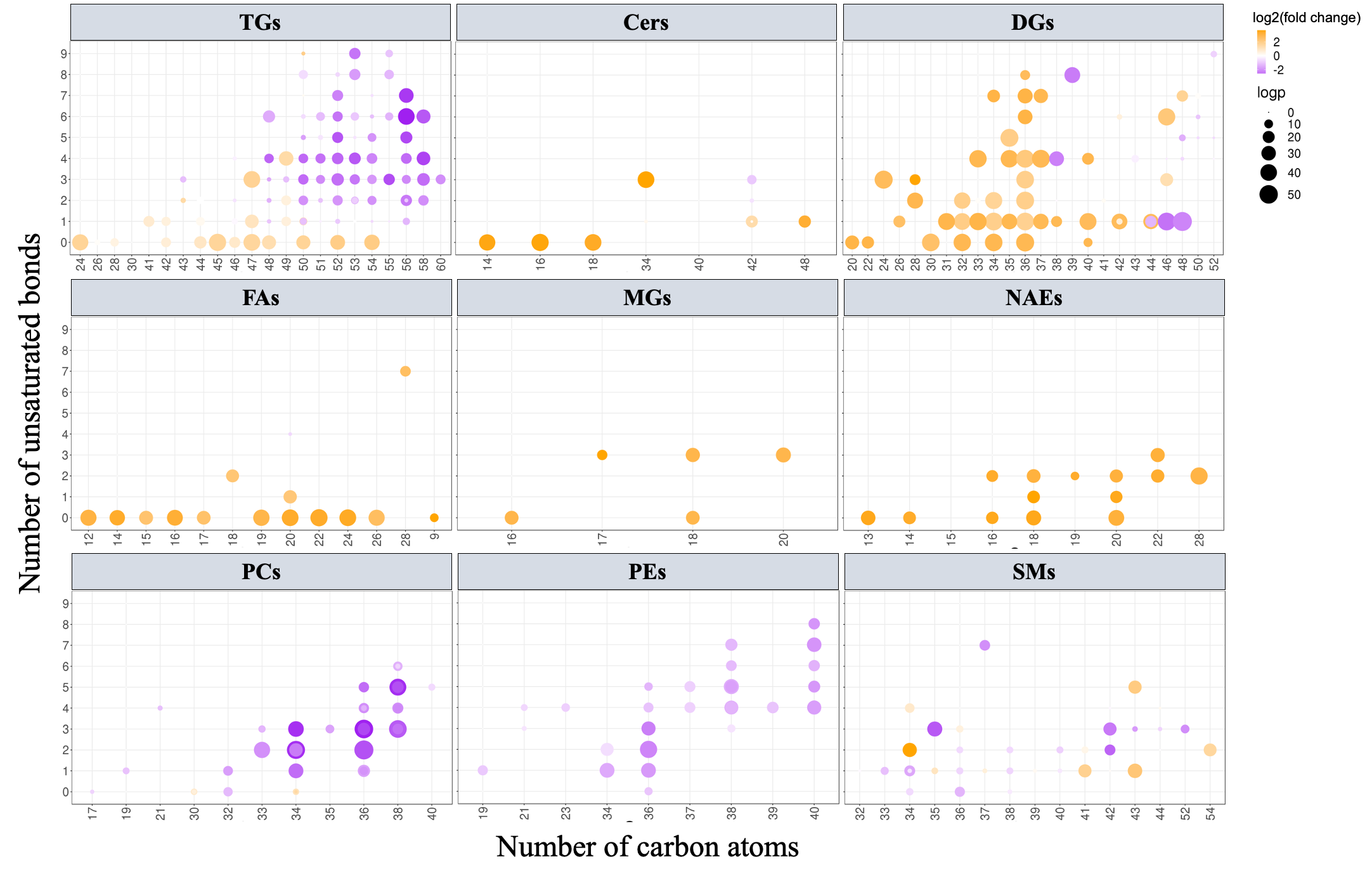


**Supplementary Figure 11.** Distinct lipidomic profiles between ICAD-D and ICAD-R. (A) Two-dimensional PCA (principal components analysis) plot of lipid profiles of ICAD-D and ICAD-R groups. (B) The heatmap showed the top 75 differential lipid species between ICAD-D and ICAD-R groups. (C) Volcano plot of the differences in lipid profiles between ICAD-D and ICAD-R groups. The lipid correlations between lipid classes of ICAD-D (D) and ICAD-R (E) groups. Data were log-transformed, and correlations between lipids were calculated using Spearman's correlation analysis. The width of the band indicates the number of correlations, and the color indicates the direction of the correlation. The correlation coefficient cut-off was set to ≥0.2.


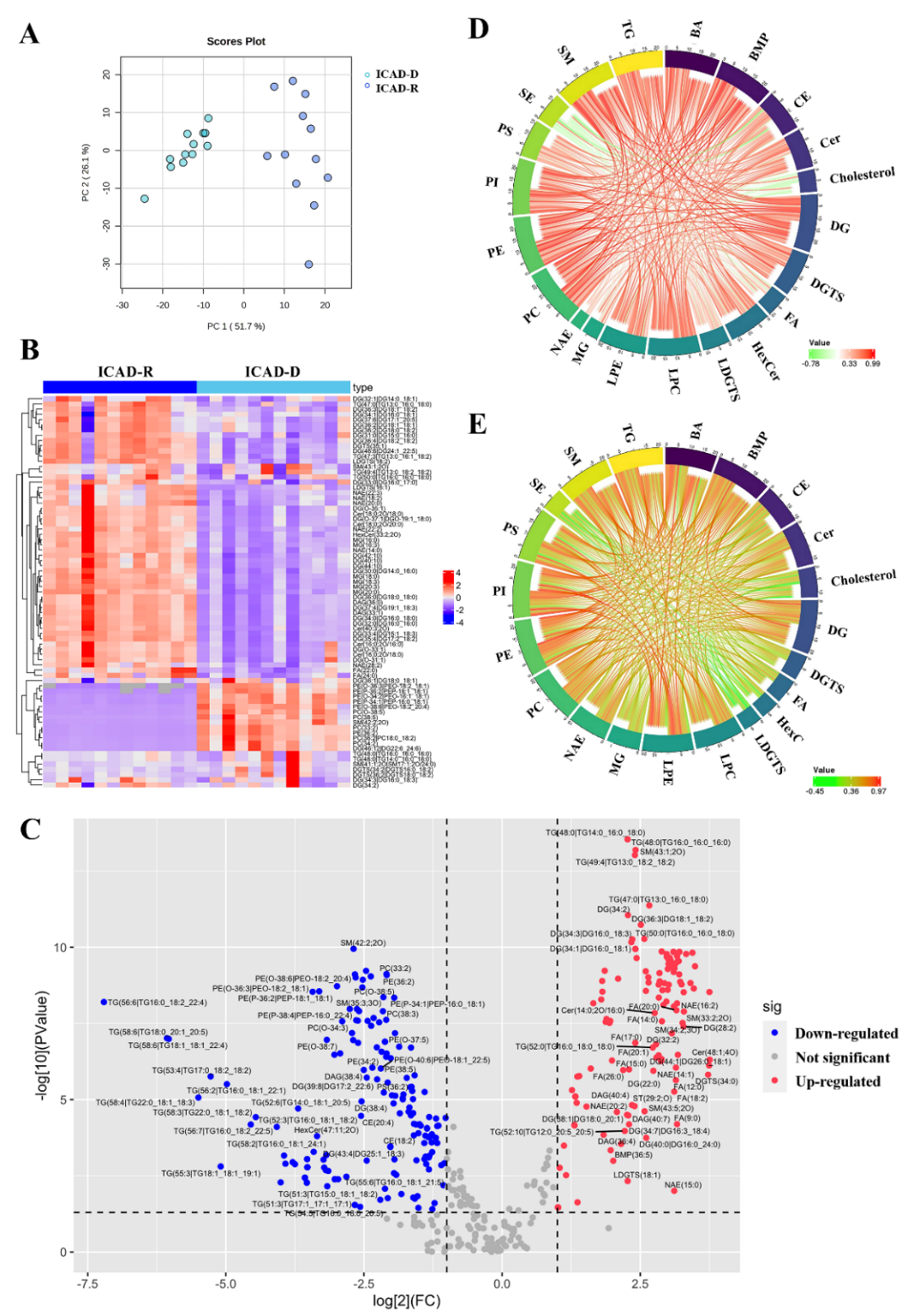


**Supplementary Figure 12.** Differential lipid species between recipient and donor cerebral vessel tissues in patients with ICAD. Lipid species within each lipid class are depicted by filled circles and arranged according to the number of total carbon atoms (x axes) and number of unsaturated bonds (y axes). The colors of the circles indicate different fold changes (recipient/donor), while the sizes of the circle indicate different ranges of P values.

**
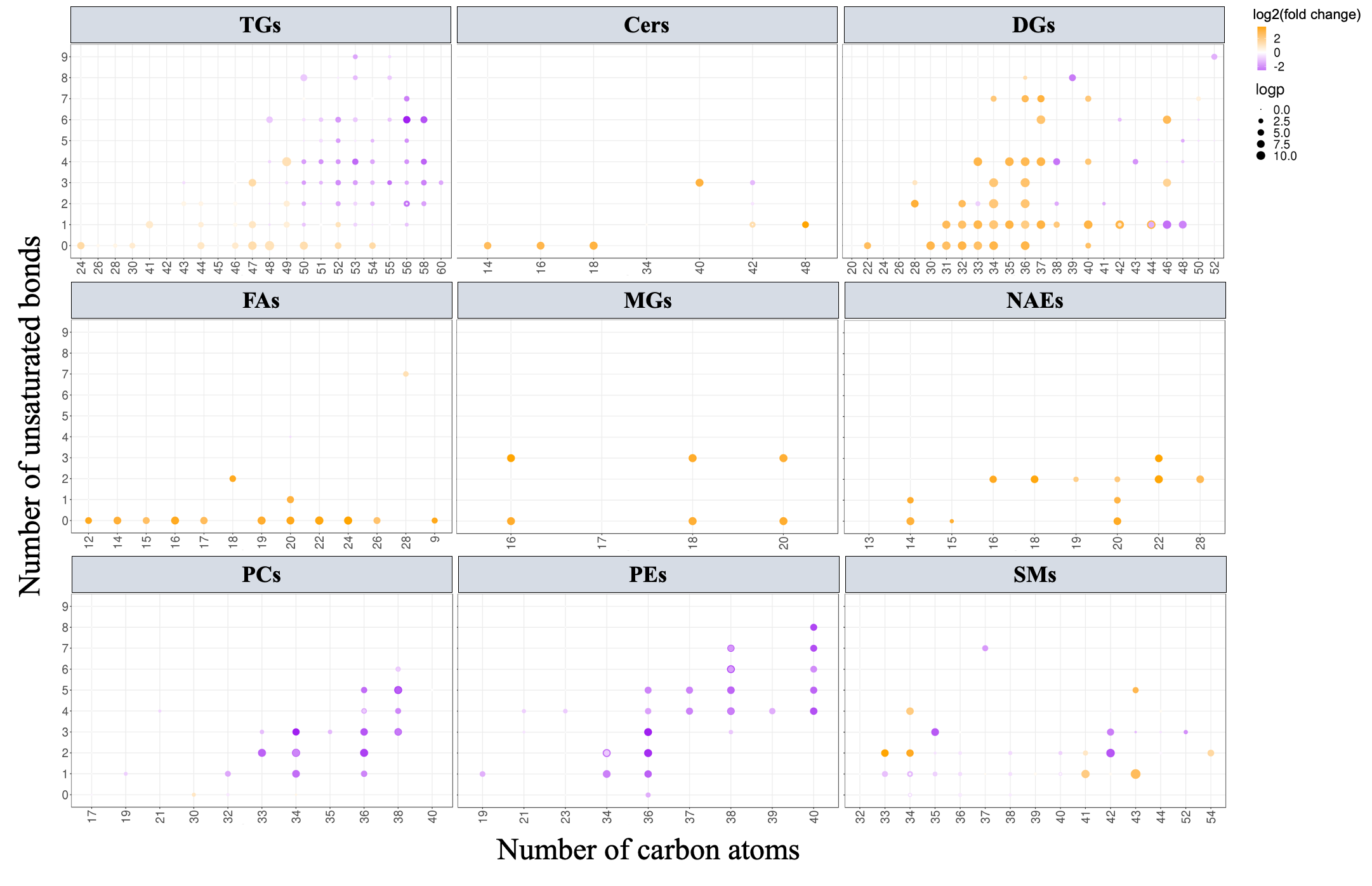
**

**Supplementary Figure 13.** Differential lipid species between recipient and donor cerebral vessel tissues. (A) Differential lipid species between recipient and donor cerebral vessel tissues in patients with MMD. (B) Differential lipid species between recipient and donor cerebral vessel tissues in patients with ICAD. Lipid species within each lipid class are depicted by filled circles and arranged according to the number of total carbon atoms (x axes) and number of unsaturated bonds (y axes). The colors of the circles indicate different fold change (recipient/ donor), while the sizes of the circle indicate different ranges of P values.


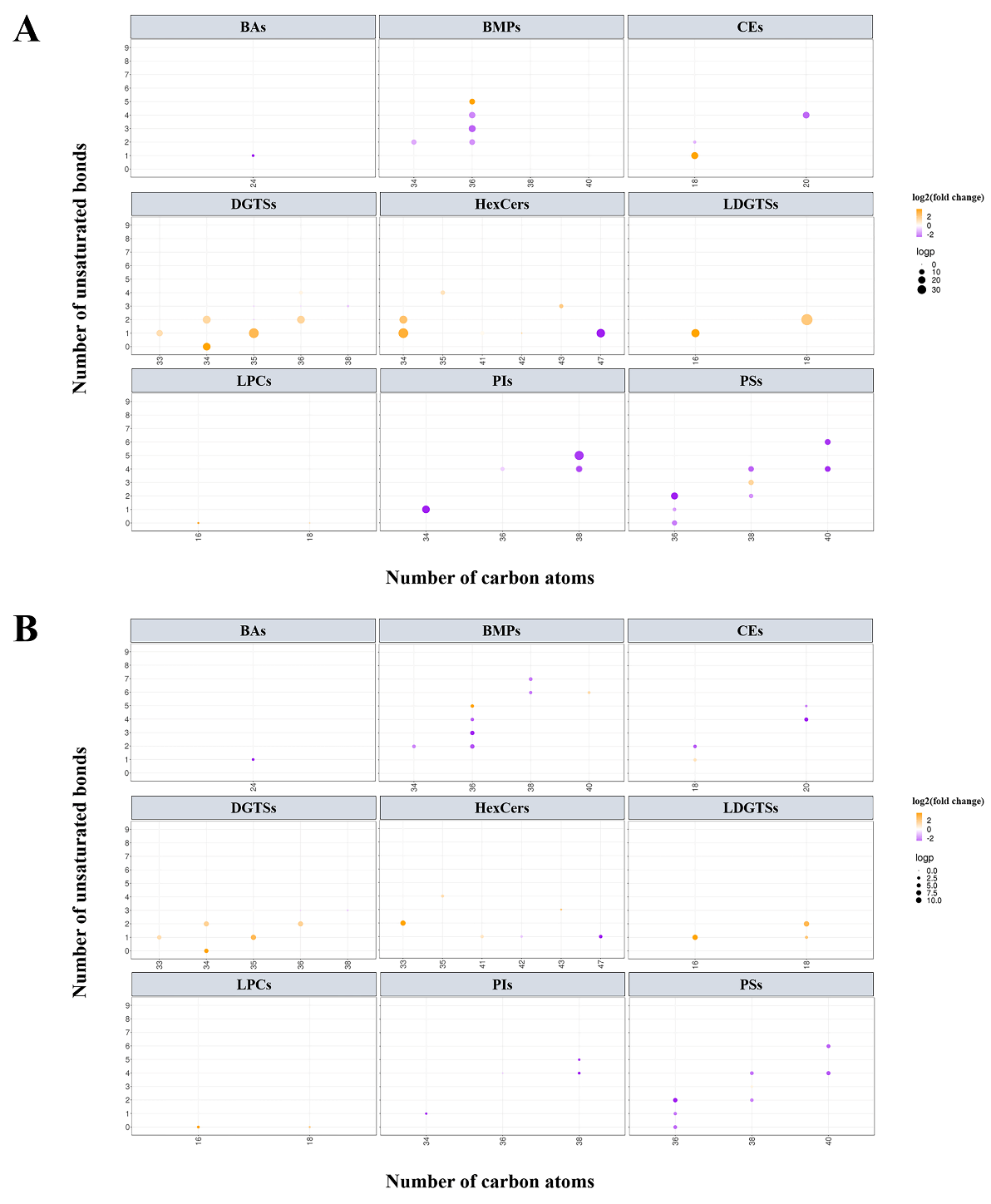


**Supplementary Figure 14.** Linear regression analysis of (A) UIBC abundance (µmol/L) and (B) TS (%) in differential lipid species.


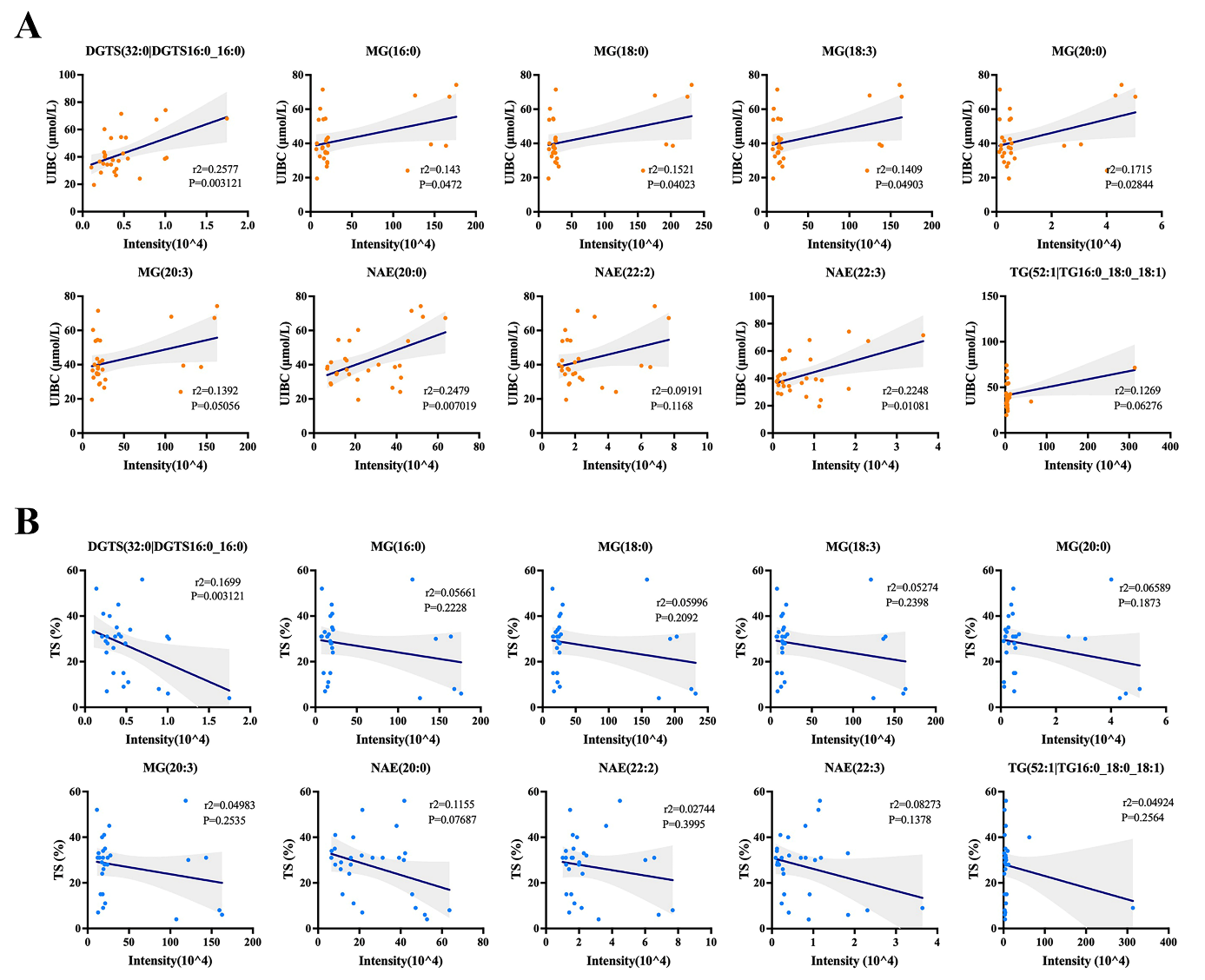


**Supplementary Figure 15.** Graphical illustration of a proposed model of disease mechanisms underlying MMD and ICAD. A lipidomics analysis to profile various lipid species in human artery tissues from patients with MMD and ICAD for discovering the novel biomarkers and mechanisms: ① A novel panel of arterial tissue lipid biomarkers with high sensitivity and accuracy in distinguishing between MMD and ICAD, as validated by ROC curve analysis. ② Significant upregulation of DG species and downregulation of TG species when comparing intracranial arteries with extracranial arteries in patients with MMD, but not in those with ICAD. ③ The top differential lipidomic biomarkers in intracranial arterial vessels exhibited negative correlations with plasma iron levels.


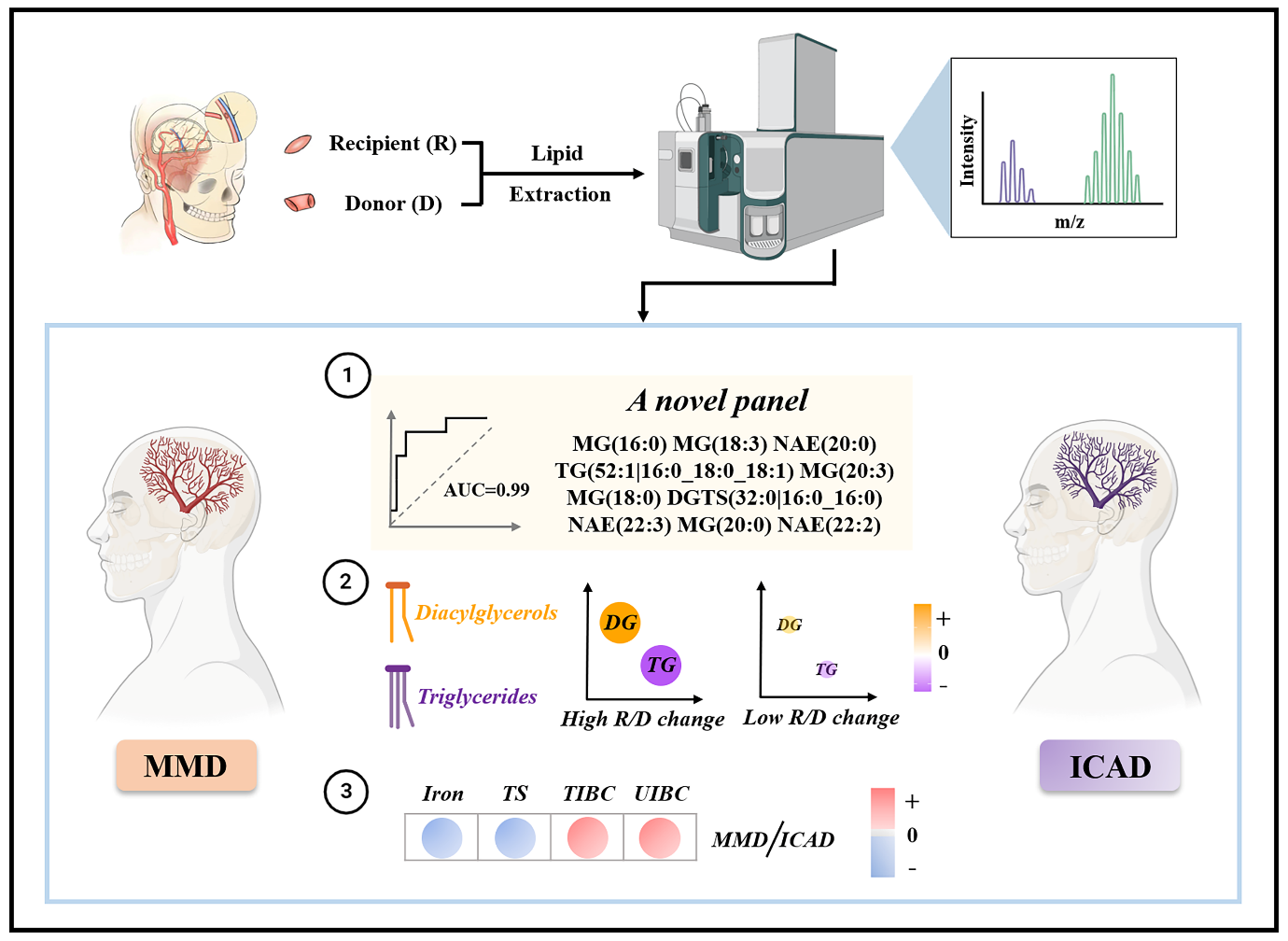


**Table S1. Demographic characteristics of the study population.**

| **Variable** | **MMD** | **ICAD** | **p value** |
| --- | --- | --- | --- |
| Number | 99 | 12 | / |
| Age, years | 44.03±0.95 | 45.08±3.18 | 0.721 |
| Male sex | 51 (48%) | 5 (42%) | 0.557 |
| Thyroid stimulating hormone, (TSH), uIU/mL | 2.69±0.22 | 2.44±0.66 | 0.641 |
| T3, ng/mL | 1.52±0.02 | 1.53±0.06 | 0.805 |
| T4, ng/mL | 77.18±1.21 | 82.57±3.59 | 0.070* |
| FT3, pg/mL | 3.14±0.03 | 2.96±0.09 | 0.021** |
| FT4, pg/mL | 13.19±0.15 | 13.38±0.47 | 0.607 |
| Anti-thyroglobulin antibodies (TG-Ab), IU/mL | 71.74±22.88 | 129.06±93.32 | 0.459 |
| Anti-thyroid peroxidase antibody (TPO-Ab), IU/mL | 71.78±22.7 | 191.69±109.5 | 0.191 |
| Total Iron binding capacity (TIBC), μmol/L | 57.24±1.85 | 60.98±1.79 | 0.0894* |
| Iron, μmol/L | 14.59±1.2 | 19.78±1.90 | 0.0208** |
| Unsaturated Iron binding capacity (UIBC), μmol/L | 44.37±2.75 | 35.66±0.73 | 0.0687* |
| Transferrin saturation degree (TS), % | 27.25±2.58 | 36.30±2.01 | 0.0178** |
| Glucose, mmol/L | 5.1±0.14 | 5.04±0.19 | 0.728 |
| Total bilirubin (TBIL), μmol/L | 10.01±0.37 | 13.27±0.88 | <0.001** |
| Direct bilirubin (DBIL), μmol/L | 2.75±0.21 | 3.06±0.24 | 0.193 |
| Total protein (TP), g/L | 67.34±0.55 | 65.53±1.51 | 0.146 |
| Albumin (ALB), g/L | 41.55±0.36 | 40.06±1.07 | 0.092* |
| ALT, U/L | 27.27±1.95 | 27.18±4.9 | 0.809 |
| AST, U/L | 21.79±0.77 | 21.74±1.8 | 0.698 |
| GGT, U/L | 27.32±2.11 | 26.24±4.95 | 0.791 |
| Prealbumin (PA), mg/L | 249.17±7.15 | 279.72±23.34 | 0.109 |
| Creatine (CREA), μmol/L | 59.6±1.31 | 65.9±2.85 | 0.012** |
| Uric acid (UA), μmol/L | 307.38±8.53 | 291.98±27.88 | 0.489 |
| Transferrin (TRF), g/L | 2.66±0.06 | 2.32±0.15 | 0.011** |
| Total cholesterol (TC), mmol/L | 4.13±0.1 | 4.34±0.32 | 0.408 |
| Triglycerides (TG), mmol/L | 1.68±0.09 | 1.6±0.22 | 0.690 |
| HDL, mmol/L | 1.04±0.03 | 1.11±0.08 | 0.272 |
| LDL, mmol/L | 2.74±0.08 | 2.82±0.24 | 0.656 |
| HDL/LDL | 9.85±9.38 | 0.42±0.05 | 0.158 |
| Lipoprotein(a) (LPa), mg/L | 208.8±23.99 | 127.82±42.08 | 0.032** |
| Homocysteine (Hcy), μmol/L | 14.39±1.17 | 16.53±4.55 | 0.551 |
| Red blood cell count (RBC), 10^12/L | 4.91±0.35 | 4.56±0.18 | 0.207 |
| White blood cell count (WBC), 10^9/L | 6.11±0.2 | 6.23±0.32 | 0.686 |
| Hemoglobin (HGB), g/L | 132.79±2.01 | 138.4±5.18 | 0.189 |
| Neutrophil ratio (NE%), % | 59.31±1.12 | 60.51±2.3 | 0.534 |
| Monocyte ratio (MO%), % | 7.51±0.88 | 6.74±0.34 | 0.251 |
| Eosinophil ratio (EO%), % | 2.25±0.14 | 1.94±0.35 | 0.293 |
| Basophil ratio (BASO%), % | 1.44±0.95 | 0.41±0.03 | 0.126 |
| Neutrophil count (NE#), 10^9/L | 4.44±0.7 | 3.8±0.28 | 0.231 |
| Lymphocyte count (LY#), 10^9/L | 2.1±0.3 | 1.87±0.14 | 0.327 |
| Monocyte count (MO#), 10^9/L | 0.47±0.07 | 0.95±0.48 | 0.204 |
| Eosinophil count (EO#), 10^9/L | 10.29±9.21 | 0.26±0.14 | 0.126 |
| Basophil count (BASO#), 10^9/L | 0.04±0.01 | 0.06±0.03 | 0.417 |
| C-reaction protein (CRP), mg/L | 3.28±0.85 | 17.92±10.14 | 0.071* |
| Serum amyloid A (SAA), mg/L | 22.53±7.43 | 16.49±10.75 | 0.536 |
| HbAlc, % | 11.86±6.03 | 5.83±0.19 | 0.165 |
| D-Dimer, mg/L | 0.58±0.13 | 0.82±0.3 | 0.350 |

Data are presented as means ± SEM. * p<0.1; ** p<0.05.
